# Chemokine receptor CCR1 restrains adipose tissue remodelling via cAMP/PKA/CREB signalling and adipocyte‒macrophage crosstalk in mice

**DOI:** 10.1002/ctm2.70762

**Published:** 2026-08-06

**Authors:** Suili Cai, Mengchen Ma, Yuqin Zhu, Mengru Shi, Xiaoyan Dai, Yujie Wang, Mengru Li, Yanwen Yu, Yajiao Wang, Liang Xu

**Affiliations:** ^1^ Key Laboratory of Laboratory Medicine, Ministry of Education, Zhejiang Provincial Key Laboratory of Medical Genetics, Cixi Biomedical Research Institute, School of Laboratory Medicine and Life Sciences Wenzhou Medical University Wenzhou China

**Keywords:** adipose remodelling, cAMP/PKA/CREB pathway, C‒C chemokine receptor 1, energy expenditure, insulin resistance, obesity

## Abstract

**Background:**

Adipose tissue remodelling, encompassing white adipose tissue (WAT) browning alongside brown adipose tissue (BAT) activation, holds promise for combating obesity, yet the endogenous chemokine receptors that restrain this plasticity remain ill‐defined. Here, we uncover that C‒C chemokine receptor 1 (CCR1) restrains adipocyte remodelling and energy expenditure.

**Methods:**

A high‐fat diet (HFD) was provided to systemic and adipocyte‐specific *Ccr1* knockout mice. The CCR1‐selective antagonist BX471 was administered to HFD‐fed wild‐type (WT) mice as a preventive regimen. Energy expenditure was assessed via indirect calorimetry; BAT thermogenic activity and WAT beiging were quantified through histology and gene expressin analyses. Adipose tissue macrophage homeostasis was analysed via flow cytometry. Peritoneal macrophages were differentiated into either the M1 or M2 state, and conditioned media were applied to the adipocytes.

**Results:**

In HFD‐fed mice, *Ccr1* ablation conferred resistance to obesity and insulin resistance, coupled with enhanced WAT browning, augmented BAT thermogenesis and elevated energy expenditure. These metabolic benefits were associated with a shift towards M2 macrophage polarisation and reduced adipose tissue inflammation. Adipocyte‐specific *Ccr1* knockout recapitulated these phenotypes. BX471 administration in WT mice phenocopied the metabolic effects of *Ccr1* deficiency under HFD conditions. Mechanistically, in vitro experiments suggested that *Ccr1* loss suppressed Gαi‐dependent signalling, leading to increased intracellular cyclic adenosine monophosphate (cAMP) and subsequent protein kinase A/cAMP‐response element‐binding protein 1 (PKA/CREB) activation, thereby promoting thermogenic gene expression in adipocytes. In addition, conditioned media from M2‐polarised macrophages enhanced thermogenic gene expression in adipocytes, and this effect was further potentiated in *Ccr1*‐deficient adipocytes.

**Conclusion:**

CCR1 regulates adipose tissue remodelling and systemic energy expenditure, at least in part, through cAMP/PKA/CREB signalling and macrophage homeostasis. Pharmacological inhibition of CCR1 attenuates obesity development, suggesting a potential preventive strategy.

**Key points:**

- CCR1 expression is significantly elevated in adipose tissue of obese individuals.
- Systemic and adipocyte‐specific *Ccr1* deletion promotes fat browning via cAMP/PKA/CREB activation and M2 macrophage polarisation, protecting against obesity.
- Pharmacological blockade of CCR1 suppresses obesity progression, highlighting its potential as a novel target for metabolic disorders.

## Graphical abstract

1. CCR1 expression is significantly elevated in adipose tissue of obese individuals.
2. Systemic and adipocyte‐specific *Ccr1* deletion promotes fat browning via cAMP/PKA/CREB activation and M2 macrophage polarisation, protecting against obesity.
3. Pharmacological blockade of CCR1 suppresses obesity progression, highlighting its potential as a novel target for metabolic disorders.

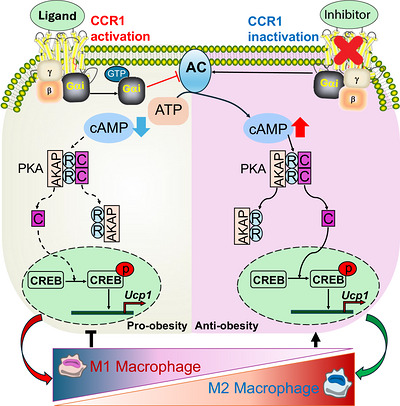

## 1 INTRODUCTION

Obesity is a growing worldwide health crisis caused by a chronic disparity between calorie consumption and expenditure, thereby causing pathological fat deposition in adipocytes. As a multifactorial metabolic disorder, obesity substantially elevates the risk of numerous chronic diseases, notably non‐alcoholic fatty liver disease, cardiovascular pathologies, type 2 diabetes and various malignancies.^1^, ^2^ Existing therapeutic interventions for obesity have limited efficacy and potential adverse effects,^3^, ^4^ highlighting the critical need for a deeper mechanistic understanding and the development of novel intervention strategies.

Mammals comprise two kinds of adipose tissue: (i) white adipose tissue (WAT), which contains white adipocytes, and (ii) brown adipose tissue (BAT), which contains brown adipocytes.^5^ WAT stores energy as triglycerides (TGs), while BAT, rich in mitochondria, burns fat via uncoupling protein 1 (UCP1) for thermogenesis.^6^ Recent studies have revealed that white adipocytes exhibit plasticity and can transform into beige adipocytes in response to factors, such as β‐adrenergic signalling, physical activity and cold challenge; this phenomenon, termed browning/beiging, resembles that of brown adipocytes in both structural and functional aspects.^7^ Beige adipocytes, prevalent in inguinal WAT (iWAT), also express UCP1 to promote energy consumption, enhance insulin sensitivity, and contribute to a new strategy for fighting obesity.^8^ Therefore, understanding the molecular mechanisms of fat browning and identifying safe and effective strategies for activating beige fat are essential to mitigating obesity and associated diseases.

Chemokines regulate both insulin resistance and obesity by inducing inflammatory cytokine production and recruiting macrophages through the G protein‐coupled receptors (GPCRs).^9^, ^10^ Additionally, recent findings indicate that chemokine‐mediated signalling modulates adipocyte differentiation and white‐fat browning.^11^ A brown adipokine, C‒X‒C motif chemokine ligand‐14 (CXCL14), triggers fat browning by inducing M2‐like macrophage polarisation,^12^ while CCL5/CCR5 signalling activation suppresses thermogenesis and browning.^13^ These findings suggest chemokines are crucial regulators of energy homeostasis and adipose browning. Nevertheless, more research is necessary to fully understand the mechanisms underlying their regulatory activities.

CCR1, a receptor for CCL3, CCL5 and CCL23, is expressed in immune cells, and is also implicated in metabolic diseases.^14^ We previously found that *Ccr1* deficiency alleviates non‐alcoholic steatohepatitis (NASH) by shifting macrophages to an anti‐inflammatory M2 phenotype.^15^ Notably, WAT of humans and rodents with obesity shows elevated CCR1 expression along with its ligands.^16^, ^17^ Nevertheless, CCR1's specific function in obesity remains elusive. Therefore, we aimed to investigate the contribution of CCR1 to the pathologic process of obesity, along with its fundamental mechanisms, and translational potential. Our findings demonstrate that both genetic deletion and pharmacological inhibition of CCR1 attenuate diet‐induced obesity (DIO) by promoting WAT browning and enhancing BAT thermogenic activity in mice.

## 2 MATERIALS AND METHODS

### 2.1 Animal experiments

Wild‐type (WT) C57BL/6 mice (SLAC Laboratory Animal Co.) and *Ccr1* knockout (*Ccr1^‒^* ^/^ *^‒^*) mice (Cyagen Biosciences) were used in this study; all animals were male and aged 7 weeks. Mice were acclimated to standard housing conditions for 1 week before randomisation into four groups (*n* = 6–8/group) and fed either a normal chow diet (NC, 10% kcal from fat, #D12450B, Research Diets) or a high‐fat diet (HFD, 60% kcal from fat, #D12492, Research Diets) for 8 weeks. Adipocyte‐specific *Ccr1* knockout (*Ccr1^AKO^*) mice and their control littermates (*Ccr1^fl/fl^*) were generated by Cyagen Biosciences. *Ccr1^AKO^* and *Ccr1^fl/fl^* mice (*n* = 5–6/group) were maintained on an HFD for 10 weeks. For prevention studies, WT mice (*n* = 7–8/group) were administered BX471 (20 mg/kg/day, i.p.), a selective CCR1 inhibitor, or vehicle during HFD feeding, as previously described.^15^ Weekly body weight measurements were taken throughout the study.

For the cold‐induced thermogenesis study, 10‐week‐old male WT and *Ccr1^‒^* ^/^ *^‒^* mice (*n* = 4/group) were transferred from 22°C (room temperature) to 4°C (a cold chamber) and exposed for 12 h without prior acclimatisation, while maintaining ad libitum water and food availability. We used a microprobe thermometer (Physitemp Instruments) to record their rectal temperature every 4 h during the cold challenge.

### 2.2 Human adipose tissue gene expression analysis

Widely obtainable GSE110729 and GSE25402 datasets were acquired from the Gene Expression Omnibus (GEO) database to analyse *CCR1* expression in human adipose tissue. GSE110729 includes abdominal subcutaneous WAT (scWAT) biopsies from 13 obese and 15 lean female subjects. GSE25402 contains abdominal scWAT samples from 30 obese and 26 lean female subjects.

### 2.3 Indirect calorimetry

Prior to the onset of significant body weight changes, the animals were housed separately in indirect calorimetry chambers (Oxymax; Columbus Instruments) for metabolic assessment. Following a 3‐day acclimation phase, we documented carbon dioxide production (VCO_2_), oxygen consumption (VO_2_) and respiratory exchange ratio (RER) for 2 uninterrupted days. Energy expenditure was computed as follows: VO_2_ × [3.815 + (1.232 × RER)], and analysed using ANCOVA, with lean mass and body weight as covariates to adjust for differences in body composition.

### 2.4 Body composition determination

Before euthanasia, an MRI apparatus (EchoMRI) was used to measure the mice's body composition.

### 2.5 Histology and immunohistochemistry

BAT, iWAT, epididymal WAT (eWAT) and liver were fixed in paraffin, and their sections underwent haematoxylin and eosin (H&E) staining or immunostaining for CD11c (1:200, #97585, Cell Signalling Technology), F4/80 (1:200, #70076, Cell Signalling Technology) or UCP1 (1:200, ab155117, Abcam), as previously described.^18^, ^19^ For immunofluorescence staining,^20^ we fixed and incubated frozen eWAT sections with anti‐CCR1 (1:100, NB100‐56334, Novus) and anti‐F4/80 (1:200, ab6640, Abcam) antibodies, and used a NIKON A1 confocal microscope (Nikon) to capture photographs. The ImageJ software (version 1.8.0; National Institutes of Health) assisted in positive area quantification.

### 2.6 Flow cytometry analysis

iWAT cells were harvested as mentioned before.^21^ We incubated the cells with Fc‐Block (BD Biosciences) and fluorochrome‐conjugated antibodies sequentially (Table S1). A FACSAria II (BD Biosciences) was used for flow cytometry, and the FlowJo software (Tree Star) was used for data analysis. We detected total macrophages as PI^−^‐CD45^+^‐NK1.1^−^‐CD3^−^‐CD19^−^‐TER119^−^‐Gr1^−^‐CD11b^+^‐F4/80^+^. Additionally, F4/80^+^‐CD11c^+^‐CD206^−^ or F4/80^+^‐CD11c^−^‐CD206^+^ cells corresponded to M1‐ or M2‐like macrophages, respectively.

### 2.7 Glucose and insulin tolerance tests

For the glucose tolerance test (GTT), mice that had fasted for 12 h received intraperitoneal glucose (2 g/kg body weight). For the insulin tolerance test (ITT), mice that had fasted for 4 h received intraperitoneal human insulin (1 U/kg body weight). We collected blood samples from the tail vein at 0, 30, 60, 90 and 120 min.

### 2.8 RNA isolation and RT‐qPCR

TRIzol reagent (Invitrogen) was used for total RNA isolation from the cells and tissues; a SuperScript II Reverse Transcriptase kit (Invitrogen) facilitated reverse transcription into cDNA per the manufacturer's specifications. We used the SYBR Green master mix (Applied Biosystems) along with the CFX384 Touch Real‐Time PCR Detection System (Bio‐Rad) for quantitative RT‐PCR. Gene expression was standardised to *β‐actin* or *18S*, and the 2^‒ΔΔCt^ technique yielded the fold change. Table S2 shows the primer sequences.

### 2.9 Biochemical analyses

We detected TG, aspartate aminotransferase (AST), glucose, non‐esterified fatty acid (NEFA), insulin, total cholesterol (TC) and alanine aminotransferase (ALT) levels using biochemical assay kits supplied by Nanjing Jiancheng Bioengineering Institute. An ELISA kit (R&D Systems, #KGE012) was used to detect cyclic adenosine monophosphate (cAMP) levels in plasma and iWAT.^22^

### 2.10 Western blot analysis

We used RIPA buffer (EMD Millipore) that contains phosphatase and protease inhibitors (Sigma‒Aldrich) to extract total proteins and conducted the BCA assay to quantify them. SDS‒PAGE separated identical protein quantities, and PVDF membranes were used for protein transfer. At 4°C, we incubated the membranes throughout the night with the following antibodies: P38 MAPK (1:1000, #9212, Cell Signalling Technology), ERK1/2 (1:1000, #9102, Cell Signalling Technology), AKT (1:1000, #9272, Cell Signalling Technology), phospho‐P38 MAPK (1:1000, #9211, Cell Signalling Technology), phospho‐ERK1/2 (1:1000, #9101, Cell Signalling Technology), UCP1 (1:1000, ab155117, Abcam), phospho‐AKT (1:1000, #9271, Cell Signalling Technology), PKA catalytic subunit (PKA‐C, 1:1000, #4782, Cell Signalling Technology), cAMP‐response element‐binding protein 1 (CREB, 1:1000, #9197, Cell Signalling Technology), phospho‐PKA‐C (1:1000, #4781, Cell Signalling Technology), phospho‐CREB (1:1000, #9198, Cell Signalling Technology) and β‐actin (1:5000, #A5441, Sigma‒Aldrich) or α‐tubulin (1:2000, #2144, Cell Signalling Technology). Chemiluminescence (Bio‐Rad) assisted in protein detection, and an imaging system (ImageLab, version 3.0, Bio‐Rad) allowed for visualisation. ImageLab software assisted in measuring band intensities, which were standardised to loading controls (β‐actin, α‐tubulin or total protein), and expressed as relative fold changes compared to the control group.

### 2.11 Pre‐adipocyte extraction and differentiation from iWAT

We used iWAT of *Ccr1^‒^* ^/^ *^‒^* and WT mice (all 10 weeks old) to prepare stromal vascular fractions (SVFs).^20^ SVF cells that attained confluence were stimulated using a differentiation medium that contained DMEM/F‐12 in addition to 20 nmol/L insulin, 10% foetal bovine serum (FBS), 5 µmol/L dexamethasone, 1 nmol/L T3, 125 µmol/L indomethacin, 500 µmol/L isobutylmethylxanthine and .5 µmol/L rosiglitazone (all from Sigma‒Aldrich) for 2 days. For 5 days, these cells were retained in a differentiation medium (DMEM/F‐12 that contains 20 nmol/L insulin, 10% FBS and 1 nmol/L T3) and replaced the medium every 2 days. Lipid accumulation was assessed by Oil Red O staining.

### 2.12 Macrophage‒adipocyte crosstalk assays

We separated peritoneal macrophages from *Ccr1^‒^* ^/^ *^‒^* and WT animals via peritoneal lavage using cold phosphate‐buffered saline (PBS).^21^ We collected the centrifuged cells and plated them in DMEM containing 10% FBS. After incubation for 2 h, the non‐adherent cells were eliminated and adherent macrophages were used for ensuing tests. For polarisation assays, we treated the macrophages with 10 ng/mL interleukin‐4 (IL‐4) or 100 ng/mL lipopolysaccharide (LPS) for 16 h. Tumour necrosis factor‐alpha (TNF‐α) and IL‐10 levels in the culture supernatants were measured via commercial ELISA kits (Nanjing Jiancheng Bioengineering Institute) as specified by the manufacturer.

For conditioned medium (CM) preparation, supernatants from LPS‐ or IL‐4‐stimulated macrophages were collected, centrifuged to eliminate debris, and passed through a .22 µm membrane. We applied the CM at a 1:1 ratio (v/v) to in vitro differentiated primary adipocytes derived from the iWAT SVF of *Ccr1^‒^* ^/^ *^‒^* and WT mice. Adipocytes were incubated with CM for 24 h and subsequently harvested for protein expression analyses.

## 3 3T3‐L1 ADIPOCYTE DIFFERENTIATION AND TREATMENT

We cultivated 3T3‐L1 preadipocytes and differentiated them as described before.^18^ Fully differentiated adipocytes were serum‐starved overnight prior to treatment. Differentiated adipocytes were treated with CCL23 (100 ng/mL), BX471 (8 µM), forskolin (8 µM), H89 (10 µM) or pertussis toxin (PTX, 100 ng/mL) for 48 h.

### 3.1 RNA sequencing and data analysis

RNA sequencing (RNA‐seq) was performed using iWAT obtained from *Ccr1^‒^* ^/^ *^‒^* and WT mice (n = 3 per group) that received an HFD. RNA‐seq as well as data analysis were commissioned by LC‐Biotechnology Co., as previously described.^15^ The GEO database (GSE274417) contains all sequencing data. Volcano plots were used to analyse the differentially expressed genes (DEGs). We used R based on the hypergeometric distribution for enrichment analyses of both the Kyoto Encyclopedia of Genes and Genomes (KEGG) and the Gene Ontology (GO) pathways.

### 3.2 Immunoprecipitation

To assess Gαi activation in adipocytes, 1 × 10^7^ serum‐starved WT and *Ccr1^‒^* ^/^ *^‒^* iWAT‐derived adipocytes (6 h) were lysed via sonication in ice‐cold immunoprecipitation buffer that contained both phosphatase inhibitors and a protease inhibitor cocktail (Santa Cruz Biotechnology). For immunoprecipitation, we incubated the lysates with an anti‐active Gαi antibody at 4°C throughout the night; 5 µL protein A Dynabeads (Thermo Fisher Scientific) was added for 1 h of culture at room temperature. Immunoprecipitated complexes were eluted using non‐reducing SDS‒PAGE loading buffer.

### 3.3 Statistical analysis

GraphPad Prism 9 (GraphPad Software Inc.) assisted in all analyses. Results are reported as mean ± SEM. To compare two groups, we used two‐tailed Student's *t*‐tests to determine significance. After one‐way or two‐way ANOVA (ordinary or repeated measures, as applicable), we used Sidak's or Tukey's post hoc tests to compare many groups. Each figure legend shows the sample sizes (*n*). Significance was set to *p* < .05.

## 4 RESULTS

### 4.1 Systemic *Ccr1* deficiency ameliorated the metabolic phenotype in DIO mice

Bioinformatic analysis of GSE110729 and GSE25402 datasets, both obtained from scWAT of female subjects, revealed significant *CCR1* upregulation in scWAT of obese humans (Figure S1A,B). To validate these findings, WT mice received 10 weeks of HFD, and RT‑qPCR assessment of adipose tissues showed elevated *Ccr1* mRNA levels in WAT as well as BAT (Figure S1C). In human scWAT (GSE25402), *CCL3* was the most consistently upregulated CCR1‐associated chemokine, whereas CCR2 and CCR5 were unchanged (Figure S1D). In mouse eWAT, multiple CCR1 ligands including *Ccl2*, *Ccl3* and *Ccl5* were significantly elevated under HFD conditions (Figure S1E). Further investigation into the cellular origin of *Ccr1* in adipose tissue revealed that lean mice showed higher *Ccr1* mRNA expression in the SVF, compared with adipocytes, whereas in DIO mice, expression levels in the SVF were comparable to those in adipocytes (Figure S1F). HFD feeding increased *Ccr1* expression in both the SVF and adipocytes (Figure S1F). Furthermore, immunofluorescence staining revealed significantly higher CCR1 expression in eWAT F4/80^+^ macrophages of DIO mice, compared with lean mice (Figure S1G). Collectively, these findings indicate increased *CCR1* expression in adipose tissues under obese conditions.

**FIGURE 1:**
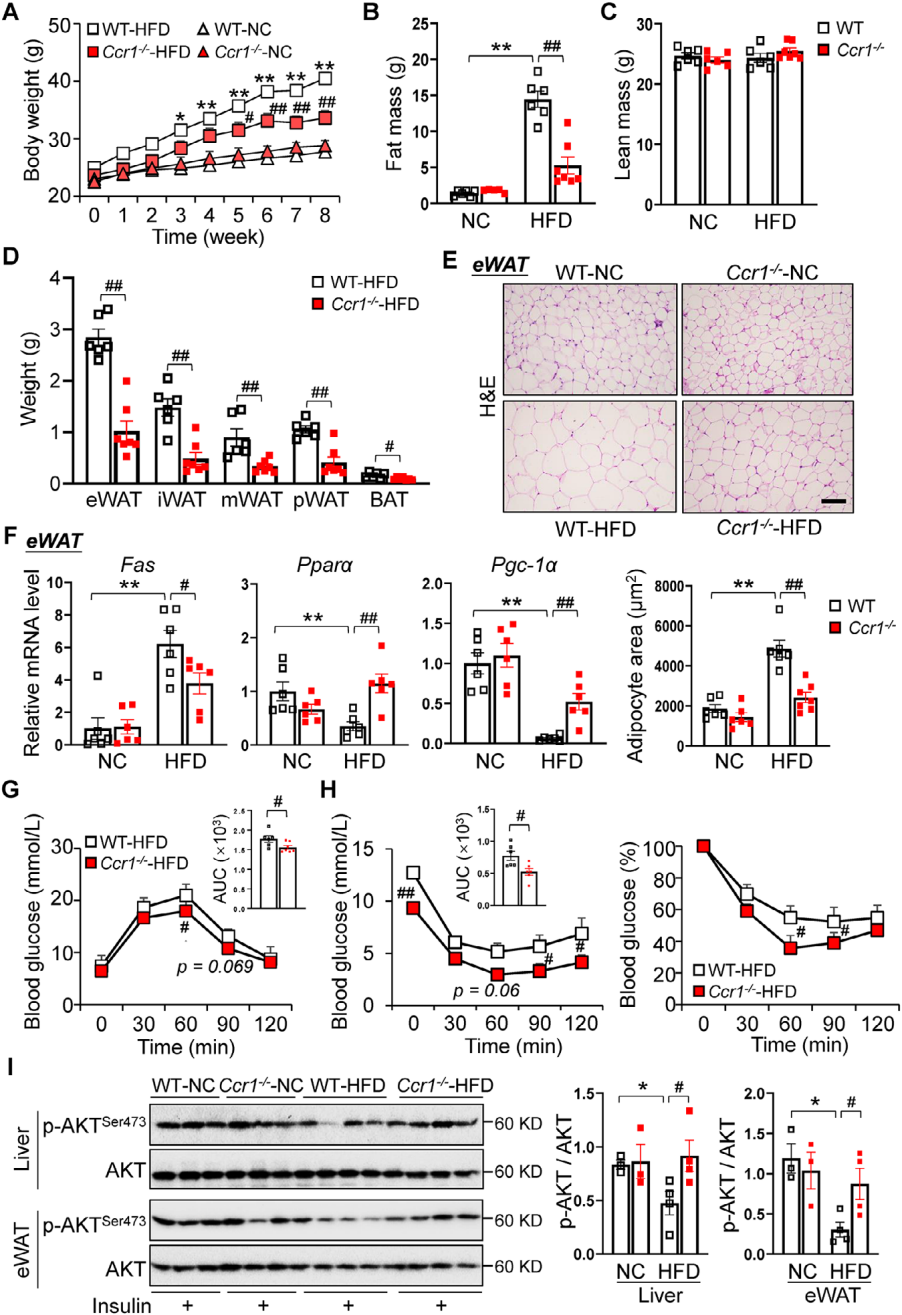
Deficiency of *Ccr1* suppresses the weight gain and insulin resistance of mice fed a high‐fat diet (HFD). (A) Body weights of mice (*n* = 6‒8). (B and C) Fat and lean mass of mice (*n* = 6‒8). (D) Tissue weights of mice (*n* = 6‒8). (E) haematoxylin and eosin (H&E)‐stained epididymal white adipose tissue (eWAT) sections (scale bars = 100 µm) and adipocyte area (*n* = 6‒8). (F) mRNA levels of *Fas*, *Pparα* and *Pgc‐1α* in eWAT of mice (*n* = 6‒8). (G and H) Glucose and insulin tolerance tests (*n* = 6‒8). (I) Immunoblots of phosphorylated Ser473 AKT (p‐AKT) and total AKT in the liver and eWAT (*n* = 3‒4). Data are presented as mean ± SEM. (A, D, G, H) Two‐tailed Student's *t*‐test. (B, C, E, F, I) Two‐way analysis of variance. ^*^ *p* < .05, ^**^ *p* < .01; ^#^ *p* < .05, ^##^ *p* < .01.

Next, the contribution of CCR1 to obesity was investigated by using *Ccr1^‒^* ^/^ *^‒^* mice. Successful deletion of *Ccr1* was first confirmed by RT‐qPCR, which showed that *Ccr1* mRNA was nearly undetectable across multiple metabolic tissues, indicating efficient global knockout (Figure S2A). In WT mice, an HFD for 8 weeks robustly promoted obesity, with a significant rise in body weight and fat mass versus NC controls (Figure 1A,B). In NC‐fed mice, *Ccr1* deficiency failed to significantly influence gain in body weight (Figure 1A), fat mass (Figure 1B), lean mass (Figure 1C), fat weight (Figure S2B), insulin and plasma lipid contents, or blood glucose levels (Table 1), indicating that the knockout did not compromise growth under energy‐balanced conditions. In contrast, *Ccr1* deficiency markedly attenuated body weight (Figure 1A), fat mass (Figure 1B) and fat weight (Figure 1D) of HFD‐fed mice. Moreover, *Ccr1* deficiency significantly lowered plasma lipid contents in DIO mice (Table 1). H&E staining revealed that *Ccr1* deletion suppressed adipocyte hypertrophy in eWAT (Figure 1E) and iWAT of DIO mice (Figure S2C). Furthermore, *Ccr1* deficiency reduced fat accumulation in BAT of DIO mice (Figure S2D) and regulated fatty acid metabolism in eWAT by decreasing *Fas* expression and increasing *Pparα* and *Pgc‐1α* mRNA levels (Figure 1F).

**TABLE 1:** Metabolic parameters in plasma of mice.

	NC		HFD	
	WT	*Ccr1^‒^* ^/^ *^‒^*	WT	*Ccr1^‒^* ^/^ *^‒^*
Plasma TG (mmol/L)	1.33 ± .10	1.02 ± .10	2.15 ± .18^**^	1.57 ± .048^##^
Plasma TC (mmol/L)	6.47 ± .79	6.88 ± .69	14.89 ± .66^**^	9.42 ± .98^##^
Plasma NEFA (mmol/L)	.17 ± .06	.18 ± .08	.35 ± .03^*^	.14 ± .04^#^
Blood glucose (mmol/L)				
Fed	7.33 ± .46	7.59 ± .31	9.83 ± .17^**^	8.51 ± .31^##^
Fasted	5.18 ± .18	5.78 ± .41	7.43 ± .24^**^	6.47 ± .18^##^
Plasma insulin (ng/mL)				
Fed	2.46 ± .36	2.57 ± .32	4.05 ± .49^*^	2.11 ± .19^##^
Fasted	.11 ± .01	.13 ± .02	.19 ± .02^**^	.14 ± .01^#^
Plasma AST (IU/L)	15.7 ± 5.6	14.6 ± 1.1	45.5 ± 2.3^**^	30.6 ± 2.9^#^
Plasma ALT (IU/L)	5.8 ± .4	6.4 ± 1.8	20.8 ± 1.5^**^	11.8 ± .8^##^

*Note*: Data are mean ± SEM. Shown are blood glucose and plasma insulin levels of mice fed (ad libitum) or fasted for 16 h. Triglyceride, total cholesterol, NEFA, AST and ALT levels were measured in fasting plasma.

Abbreviations: ALT, alanine transaminase; AST, aspartate transaminase; HFD, high‐fat diet; NC, normal chow diet; NEFA, non‐esterified fatty acid; TC, total cholesterol; TG, triglyceride; WT, wild type.

^*^ *p* < .05,

^**^ *p* < .01 versus NC‐fed WT mice.

^#^ *p* < .05.

^##^ *p* < .01 versus HFD‐fed WT mice.

*Ccr1* deficiency also improved metabolic parameters, reducing elevations in plasma glucose and insulin levels induced by HFD (Table 1). GTT and ITT results demonstrated that *Ccr1* loss in DIO mice ameliorated glucose intolerance as well as insulin resistance (Figure 1G,H), whereas no significant effects were observed in NC‐fed mice (Figure S2E,F). Notably, *Ccr1^‒/–^* HFD mice displayed significantly lower baseline blood glucose levels prior to insulin injection (Figure 1H). Accordingly, basal p‐AKT^Ser473^ levels were minimal in both liver and eWAT (Figure S2G,H), whereas insulin stimulation markedly increased p‐AKT^Ser473^ in both tissues, and *Ccr1^‒^* ^/^ *^‒^* mice showed a more pronounced induction (Figure 1I). Overall, these findings demonstrate that systemic *Ccr1* deficiency in HFD‐fed mice attenuates body weight increase and insulin resistance.

### 4.2 Loss of *Ccr1* enhanced energy expenditure and fat browning in DIO mice

**FIGURE 2:**
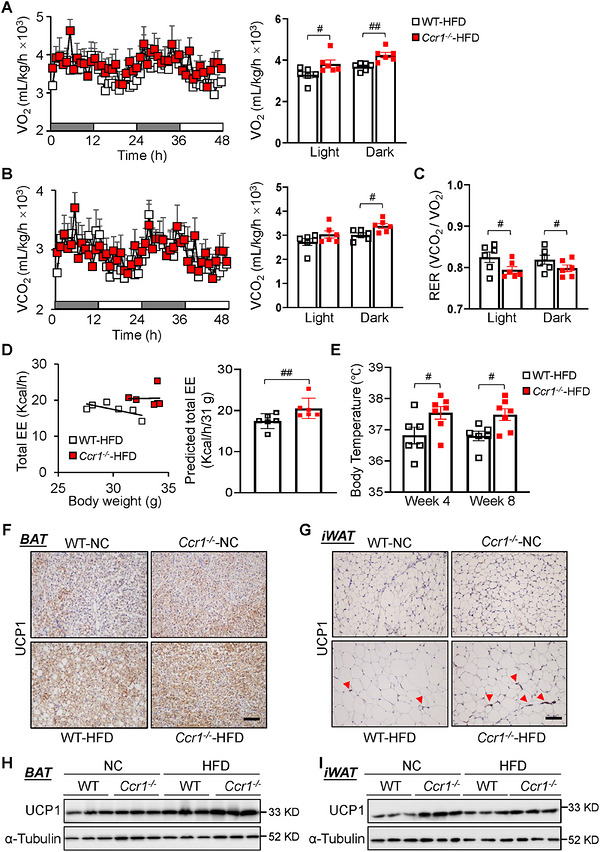
Deficiency of *Ccr1* increases energy expenditure in diet‐induced obesity (DIO) mice. (A) VO_2_ of the mice (*n* = 6). (B) VCO_2_ of the mice (*n* = 6). (C) Respiratory exchange ratio (RER) of the mice (*n* = 6). (D) Energy expenditure (EE) plotted against body weight and ANCOVA predicted energy expenditure at a given body weight of 31 g in mice (*n* = 6). The ANCOVA body weight effect is *p* = .008. (E) Rectal temperature of the mice (*n* = 6‒8). (F and G) Uncoupling protein 1 (UCP1)‐stained brown adipose tissue (BAT) and inguinal white adipose tissue (iWAT) sections in normal chow diet (NC)‐fed and high‐fat diet (HFD)‐fed mice (*n* = 6). Scale bars = 100 µm. (H and I) Immunoblots of UCP1 in BAT and iWAT of mice (*n* = 6). Data are presented as mean ± SEM. (A‒E) Two‐tailed Student's *t*‐test. ^#^ *p* < .05, ^##^ *p* < .01.

To evaluate whole‐body energy expenditure, after being fed an HFD for 4 weeks, we placed the animals in indirect calorimetry cages, before obvious body mass changes. Compared to WT mice, *Ccr1^‒^* ^/^ *^‒^* mice exhibited higher VO_2_ and correspondingly elevated VCO_2_ under both NC and HFD conditions (Figures 2A,B and S3A,B). Moreover, *Ccr1* deficiency did not significantly alter the RER in mice maintained on NC (Figure S3C), but decreased the RER in HFD‐fed mice (Figure 2C), suggesting enhanced fat utilisation under nutrient excess conditions. Furthermore, *Ccr1* knockout in DIO mice raised whole‐body energy expenditure (Figure 2D). Consistently, *Ccr1* depletion did not influence core temperature in NC‐fed mice (Figure S3D), but significantly elevated core temperature by approximately 1°C in their HFD‐fed counterparts (Figure 2E). Thus, a lack of *Ccr1* raises energy expenditure, thereby reducing obesity.

The raised body temperature and augmented energy expenditure in *Ccr1^–/–^* HFD mice imply a rise in adaptive thermogenesis. In HFD‐fed mice, *Ccr1* deficiency elevated the UCP1^+^ cell number in BAT (Figure 2F), which was accompanied by increased UCP1 protein levels (Figures 2H and S4A). Additionally, HFD‐fed *Ccr1^‒^* ^/^ *^‒^* mice showed significantly higher mRNA levels of brown fat‐selective genes, including *Prdm16*, *Dio2*, *Pparγ* and *Pgc‐1α*, in BAT (Figure S4B).

Moreover, immunohistochemical analysis revealed that *Ccr1* depletion enhanced browning in iWAT of DIO mice, with increased UCP1^+^ cells (Figure 2G), elevated UCP1 protein levels (Figures 2I and S4C), and upregulated mRNA levels of *Prdm16*, *Dio2*, *Cidea* and *Pparγ* mRNA levels (Figure S4D). Consistently, HFD‐fed *Ccr1*‐deficient mice showed increased mRNA levels of these genes in the eWAT (Figure S4E).

To explore the influence of CCR1 on adipocyte differentiation in vitro, preadipocytes were separated from the iWAT of *Ccr1^‒^* ^/^ *^‒^* and WT mice, and differentiation was induced. *Ccr1* deficiency attenuated preadipocyte differentiation in vitro (Figure S4F), increased mRNA levels of brown fat‐selective genes, and suppressed fat synthesis‐related gene expression (Figure S4G). Overall, *Ccr1* deficiency promotes BAT function and WAT browning.

### 4.3 Loss of *Ccr1* promoted cAMP/protein kinase A/CREB signalling activation in the iWAT of HFD‐fed mice

**FIGURE 3:**
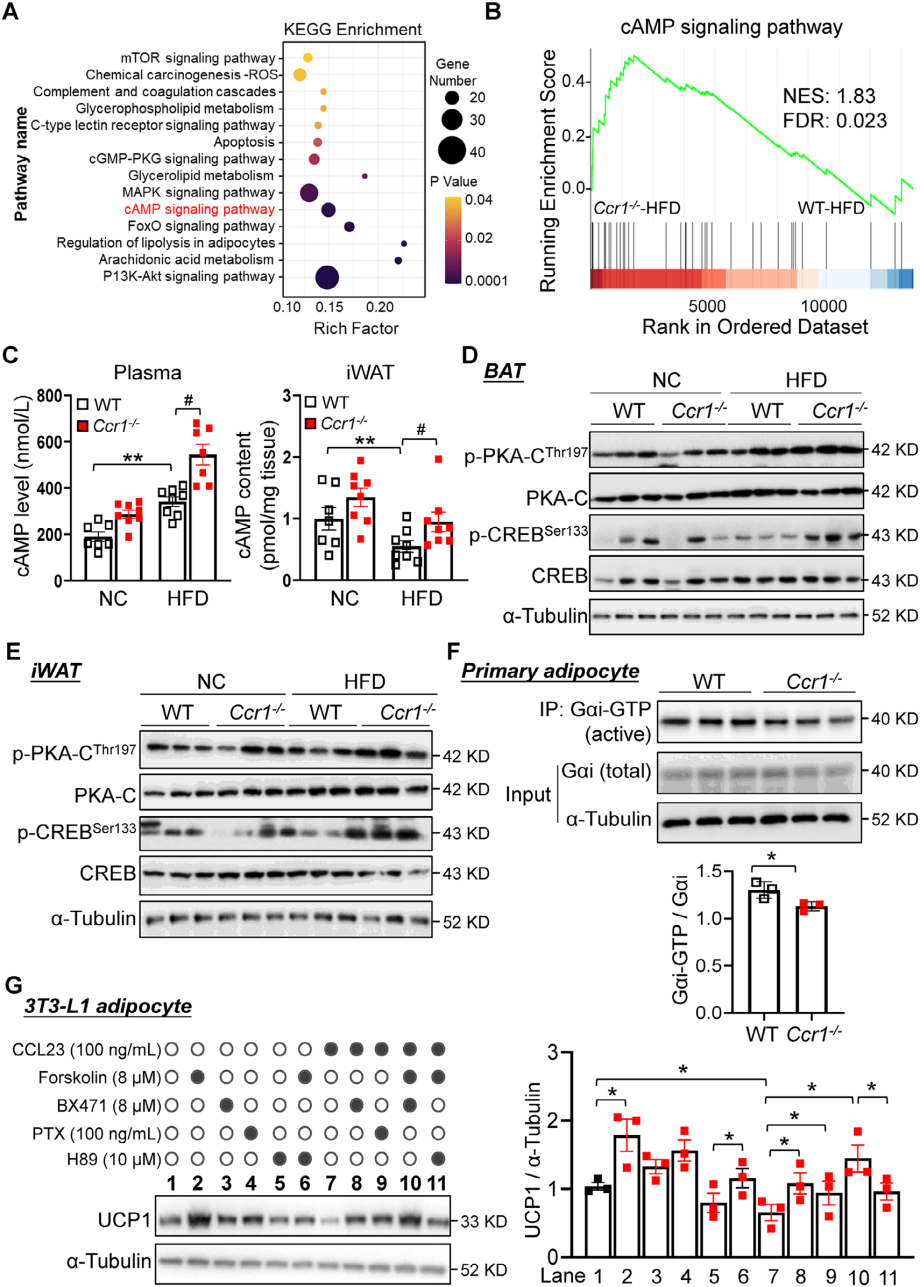
Deficiency of *Ccr1* enhances cAMP/protein kinase A (PKA)/cAMP‐response element‐binding protein 1 (CREB) pathway in both brown adipose tissue (BAT) and inguinal white adipose tissue (iWAT) of high‐fat diet (HFD)‐fed mice. (A) Kyoto Encyclopedia of Genes and Genomes (KEGG) enrichment analysis of differentially expressed genes (DEGs) in the iWAT of HFD‐fed mice (*n* = 3). (B) GSEA of cAMP pathway in the iWAT of HFD‐fed mice (*n* = 3). (C) cAMP levels in plasma and iWAT of mice (*n* = 6‒8). (D and E) Immunoblots of p‐PKA‐C and p‐CREB and their total proteins within BAT and iWAT of mice (*n* = 6‒8). (F) Immunoprecipitation analysis of Gαi in iWAT‐derived adipocytes (*n* = 3). (G) Immunoblots of uncoupling protein 1 (UCP1) in 3T3‐L1 adipocytes (*n* = 3). Data are presented as mean ± SEM. (C) Two‐way analysis of variance. (F and G) Two‐tailed Student's *t*‐test. ^*^ *p* < .05.

We next explored the mechanisms behind *Ccr1* knockout‐triggered WAT browning; thus, RNA‐seq was performed on iWAT from HFD‐fed WT and *Ccr1^‒^* ^/^ *^‒^* mice, as this depot exhibits high metabolic plasticity and strong inducibility, making it particularly suitable for capturing adaptive transcriptional responses to dietary challenge.^23^ Compared with WT controls, *Ccr1^‒^* ^/^ *^‒^* mice showed 824 downregulated DEGs and 933 upregulated DEGs (Figure S5A). As predicted, GO enrichment analysis of the DEGs pointed to their involvement in several key biological processes, ranging from cell differentiation and lipid metabolic process to heat generation and adaptive thermogenesis (Figure S5B). Moreover, the same set of genes was mapped to the PI3K‐Akt, MAPK, and cAMP signalling cascades based on KEGG pathway annotation (Figure 3A). Notably, cAMP is recognised as the core regulator of the biology of both brown and beige adipocytes and stimulates the downstream protein kinase A (PKA)/CREB signalling cascade in rodents.^22^, ^24^ Gene set enrichment analysis (GSEA) and heatmap analyses further confirmed activation of cAMP‐related signalling pathways upon *Ccr1* loss (Figures 3B and S5C). Next, cAMP levels were measured in plasma and iWAT of HFD‐fed mice and found to be significantly elevated by *Ccr1* deletion relative to WT controls (Figure 3C). Furthermore, *Ccr1* knockout raised phosphorylation levels of PKA‐C and CREB within BAT and iWAT (Figures 3D,E and S5D,E).

CCR1 suppresses adenylate cyclase activity through Gαi‐coupled signalling, thereby reducing intracellular cAMP accumulation and attenuating PKA/CREB signalling.^14^ Ablation of *Ccr1* significantly attenuated Gαi activation in primary adipocytes, as evidenced by reduced Gαi‐GTP levels (Figure 3F). In 3T3‐L1 adipocytes, a well‐established and highly tractable in vitro model for studying adipocyte biology, exposure to CCL23, a chemokine that preferentially activates CCR1,^25^ led to a marked decrease in UCP1 protein levels (Figure 3G). By contrast, application of the CCR1 antagonist BX471 produced a strong upregulation of UCP1 (Figure 3G), which was abolished by co‐treatment with H89 (a PKA inhibitor); thus, the BX471‐induced raise in UCP1 expression was dependent on PKA signalling (Figure 3G). In parallel, inhibition of Gαi signalling using PTX reversed the suppressive effect of CCL23 on UCP1 expression (Figure 3G). Concurrently, RT‐qPCR analysis corroborated these findings, showing parallel modulation of *Ucp1* and *Pgc‐1α* expression following pharmacological manipulation of this signalling axis (Figure S5F). Collectively, these findings indicate that the loss of *Ccr1* promotes adipocyte thermogenic activation, at least partially, through the cAMP‐mediated PKA/CREB cascade.

### 4.4 Adaptive thermogenesis was elevated in cold‐stimulated *Ccr1^‒^* ^/^ *^‒^* mice

**FIGURE 4:**
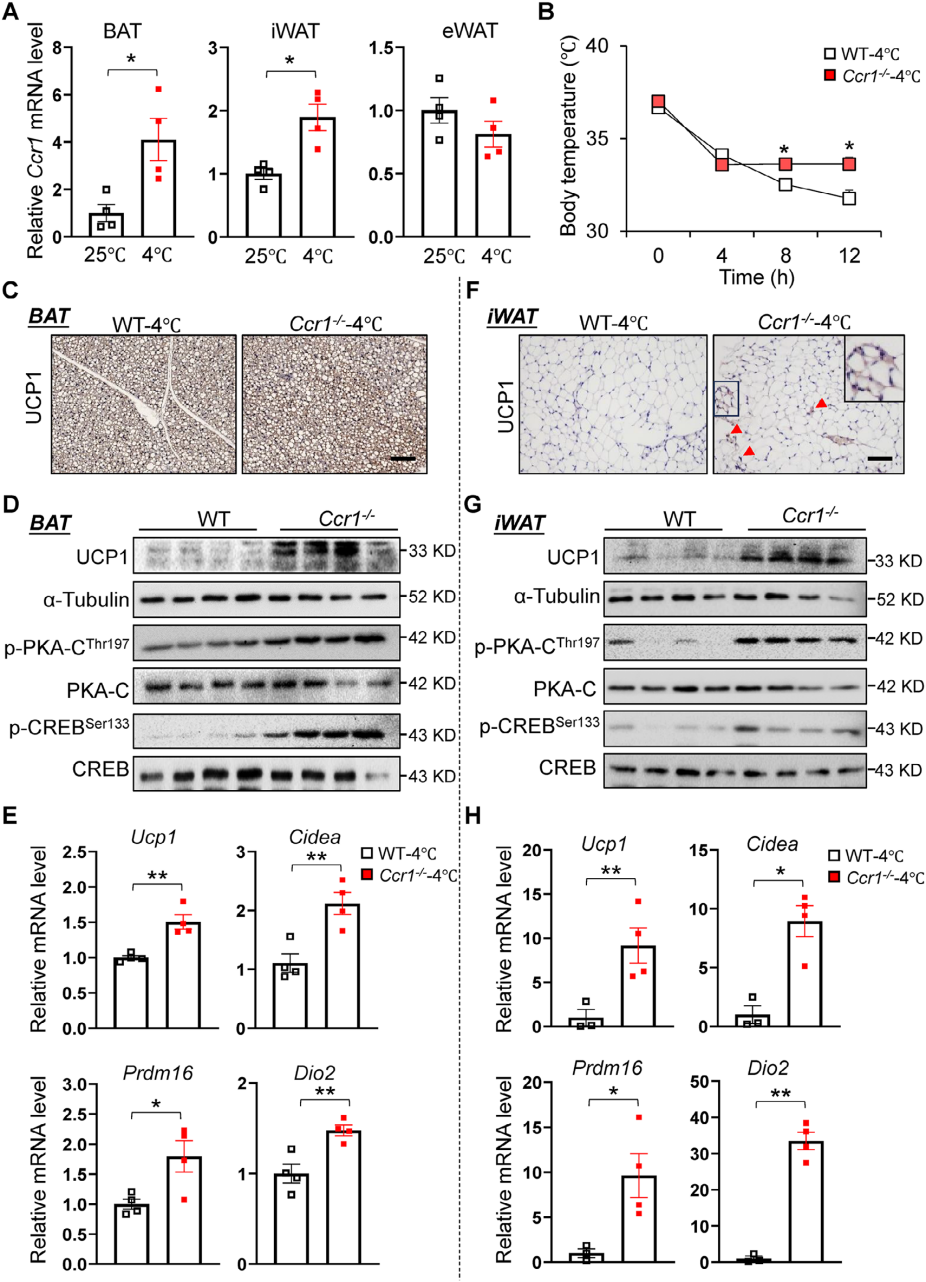
Deficiency of *Ccr1* increases adaptive thermogenesis of cold‐stimulated mice. (A) Relative mRNA expression of *Ccr1* in the adipose tissues of 4°C‐stimulated mice (*n* = 4). (B) Rectal temperature of mice (*n* = 4). (C) Uncoupling protein 1 (UCP1)‐stained brown adipose tissue (BAT) sections of mice (*n* = 4). Scale bars = 100 µm. (D) Immunoblots of UCP1, p‐PKA‐C and p‐CREB and their total proteins in BAT of 4°C‐stimulated mice (*n* = 4). (E) Relative mRNA levels of thermogenic genes in BAT of mice (*n* = 4). (F) UCP1‐stained inguinal white adipose tissue (iWAT) sections of mice (*n* = 4). Scale bars = 100 µm. (G) Immunoblots of UCP1, p‐PKA‐C and p‐CREB and their total proteins in iWAT of mice (*n* = 4). (H) Relative mRNA levels of thermogenic genes in iWAT of 4°C‐stimulated mice (*n* = 4). Data are presented as mean ± SEM. (B‒H) Two‐tailed Student's *t*‐test. ^*^ *p* < .05, ^**^ *p* < .01.

Initially, compared to normothermic conditions (22°C), *Ccr1* mRNA expression was significantly upregulated in BAT as well as iWAT under cold exposure (4°C), whereas no significant changes were detected in eWAT (Figure 4A). We further evaluated the impact of *Ccr1* knockout on whole‐body thermogenic capacity. Basal rectal temperatures were similar between WT and *Ccr1^‒^* ^/^ *^‒^* mice (WT = 36.8 ± .2°C, *Ccr1^‒^* ^/^ *^‒^* = 37.1 ± .1°C) under normothermic conditions (22°C) (Figure 4B). However, upon exposure to a 4°C environment, *Ccr1* deficiency conferred protection against the cold challenge, as evidenced by an increase in core body temperature of approximately 2°C (WT = 31.7 ± .2°C, *Ccr1^‒^* ^/^ *^‒^* = 33.4 ± .1°C) (Figure 4B), indicating enhanced cold tolerance.

Moreover, *Ccr1* deficiency raised the UCP1^+^ cell number (Figure 4C,F), accompanied by elevated UCP1 protein levels, in both BAT and iWAT of cold‐exposed mice (Figures 4D,G and S6A,B). These changes were further accompanied by augmented mRNA levels of thermogenic genes, including *Ucp1*, *Cidea*, *Prdm16* and *Dio2* in both BAT and WAT (Figures 4E,H and S6C). Strikingly, deficiency of *Ccr1* also enhanced the cAMP/PKA/CREB cascade activation under cold conditions (Figures 4D,G and S6A,B). Together, *Ccr1* depletion increases adaptive thermogenesis by promoting BAT function and fat browning under cold stress conditions.

### 4.5 Loss of *Ccr1* promoted M2 macrophage polarisation and attenuates WAT and liver inflammation

**FIGURE 5:**
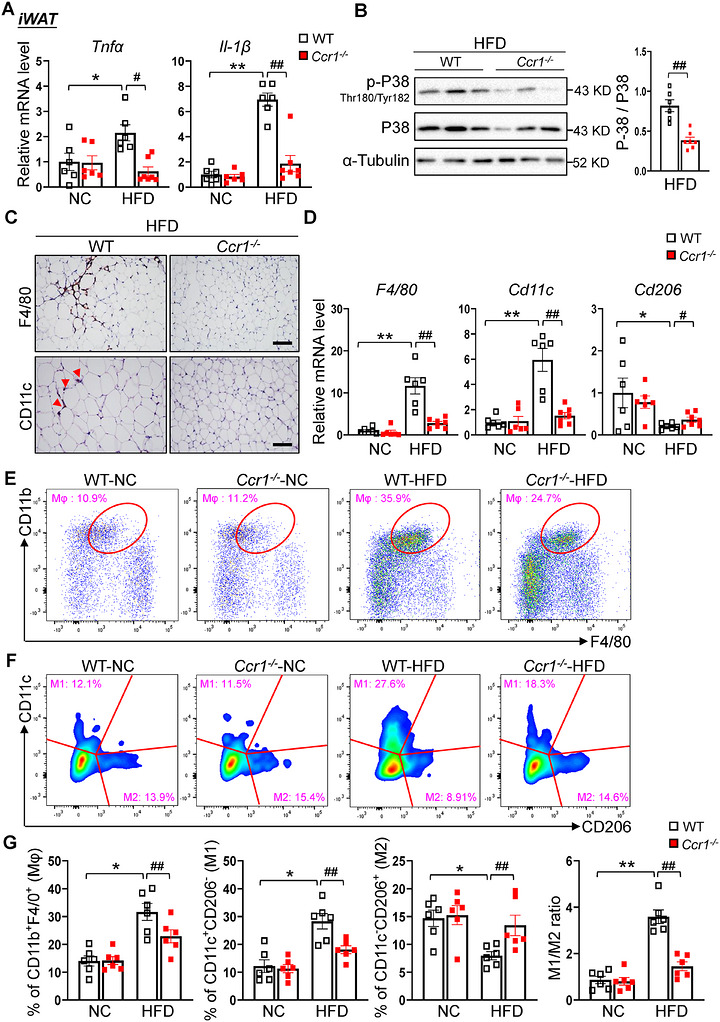
Deficiency of *Ccr1* decreases inflammation and promotes the polarisation of macrophages towards the anti‐inflammatory phenotype in inguinal white adipose tissue (iWAT) of obese mice. (A) Relative mRNA expression of *Tnfα* and *Il‐1β* in the iWAT of mice (*n* = 6‒8). (B) Immunoblot analysis of p‐P38 MAPK and P38 MAPK with iWAT lysates of high‐fat diet (HFD)‐fed mice (*n* = 6‒8). (C) F4/80 and CD11C immunostaining in iWAT sections of HFD‐fed mice (*n* = 6‒8). Scale bars = 100 µm. (D) Relative mRNA expression of *F4/80*, *Cd206* and *Il‐10* in the iWAT of mice (*n* = 6‒8). (E) Representative flow cytometry plots of total macrophages in iWAT (*n* = 6). (F) M1 and M2 macrophages of iWAT (*n* = 6). (G) Quantitation of total macrophages, M1‐ and M2‐type macrophages and M1/M2 ratio (*n* = 6). Data are presented as mean ± SEM. (A, D‒G) Two‐way analysis of variance. (B) Two‐tailed Student's *t*‐test. ^*^ *p* < .05, ^**^ *p* < .01; ^#^ *p* < .05, ^##^ *p* < .01.

*Ccr1* knockout attenuated the HFD‐induced elevation of *Tnfα* and *Il‐1β* expression (Figure 5A). This reduction was accompanied by decreased phosphorylation of p38 MAPK in iWAT (Figure 5B). Immunostaining analysis revealed a decrease in F4/80^+^ and CD11c^+^ cells in iWAT of *Ccr1* ^–/–^ HFD mice, which was accompanied by decreased *F4/80* and *Cd11c* mRNA levels (Figure 5C,D). Consistently, FACS analysis revealed *Ccr1* loss significantly attenuated the HFD‐induced accumulation of macrophages in iWAT (Figure 5E,G), and shifted the macrophage phenotype from M1 to M2 (Figure 5F,G), along with upregulation of mRNA levels of M2 markers *Cd206* (Figure 5D). Similarly, eWAT from *Ccr1*‐deficient HFD‐fed mice showed reduced macrophage infiltration and inflammatory status (Figure S7A‒E). Collectively, *Ccr1* deficiency promotes M2 macrophage polarisation within WAT, potentially contributing to adipocyte browning.

To further validate macrophage functional phenotypes, after separating peritoneal macrophages from WT and *Ccr1^‒^* ^/^ *^‒^* mice, we performed LPS or IL‐4 stimulation. ELISA analysis showed that *Ccr1* deficiency significantly reduced TNF‐α secretion upon LPS stimulation, whereas IL‐10 secretion was markedly increased following IL‐4 treatment, indicating a shift towards the anti‐inflammatory M2‐like phenotype (Figure S8A,B). To further determine whether macrophage‐derived signals directly promote adipocyte thermogenesis, CM from IL‐4‐stimulated macrophages was applied to primary adipocytes. This treatment robustly enhanced thermogenic activation, as evidenced by increased CREB phosphorylation and UCP1 protein levels; the outcome was further enhanced in adipocytes lacking *Ccr1* (Figure S8C,D), suggesting that *Ccr1* deficiency increases adipocyte responsiveness to pro‐thermogenic paracrine cues.

Additionally, *Ccr1* deficiency reduced plasma ALT and AST levels (Table 1), and attenuated hepatic lipid accumulation in HFD‐fed mice (Figure S9A,B). These results were related to lipogenic genes (*Fas* and *Scd1*) suppression and mitochondrial fatty acid β‐oxidation gene upregulation (*Cpt‐1α* and *Pparα*) (Figure S9C). Similar to the effects in WAT, *Ccr1* deficiency markedly reduced F4/80^+^ cells along with *F4/80* and *Cd11c* mRNA levels in the liver of DIO mice (Figure S9D,E). Moreover, proinflammatory cytokines and chemokines showed downregulation, and p38 MAPK and ERK phosphorylation were diminished in the liver of HFD‐fed mice (Figure S9F,G).

### 4.6 Adipocyte‐specific deficiency of *Ccr1* suppressed HFD‐induced obesity via enhancing browning

**FIGURE 6:**
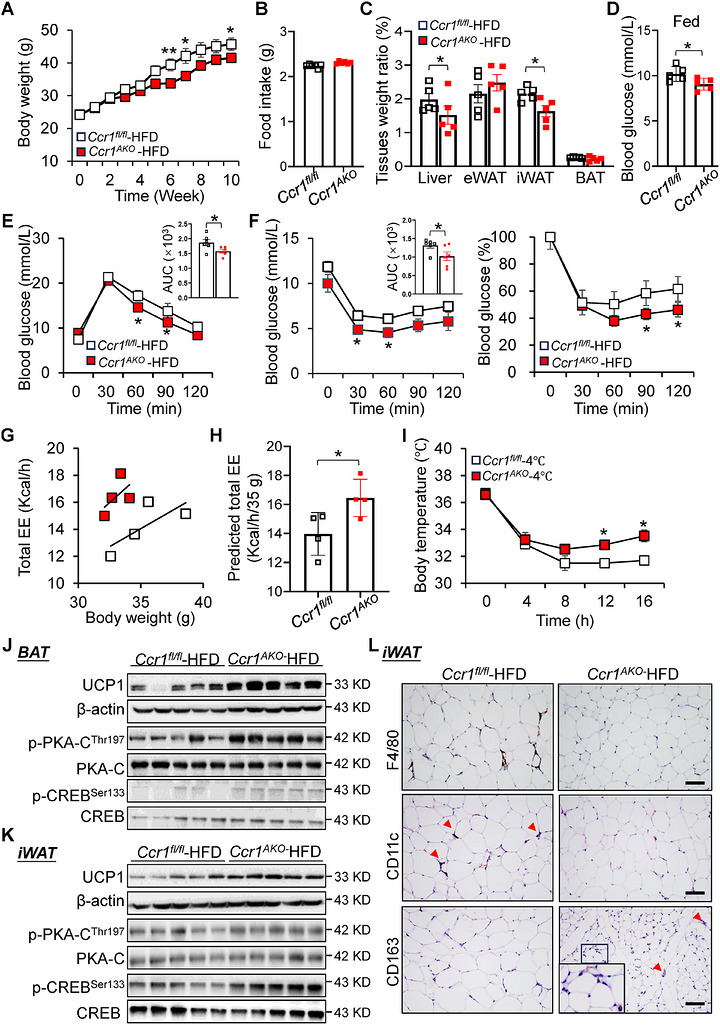
Adipocyte‐specific *Ccr1* knockout protects mice from high‐fat diet (HFD)‐induced obesity and insulin resistance. (A) Body weights of mice (*n* = 4‒5). (B) Food intake of mice (*n* = 4‒5). (C) Tissue weight ratio of mice (*n* = 4‒5). (D) Fed blood glucose levels of mice (*n* = 4‒5). (E and F) Glucose and insulin tolerance tests (*n* = 4‒5). (G and H) Energy expenditure of mice (*n* = 4). The ANCOVA body weight effect is *p* = .012. (I) Body temperature of 4°C‐stimulated mice (*n* = 4‒5). (J and K) Immunoblots of uncoupling protein 1 (UCP1), p‐PKA‐C and p‐CREB and their total proteins in brown adipose tissue (BAT) and inguinal white adipose tissue (iWAT) of mice (*n* = 5). (L) F4/80, CD11c and CD163 immunostaining in iWAT sections of HFD‐fed mice (*n* = 4‒5). Scale bars = 100 µm. Data are presented as mean ± SEM. (A‒I) Two‐tailed Student's *t*‐test. ^*^ *p* < .05, ^**^ *p* < .01.

To dissect CCR1's function in obesity development, we established adipocyte‐specific *Ccr1* deficiency in a mouse model (Figure S10A,B) and subjected these mice to HFD for 10 weeks. As expected, *Ccr1* deficiency in adipocytes significantly mitigated weight gain (Figure 6A), with minimal impact on food intake (Figure 6B). This deficiency also reduced liver and iWAT weight (Figure 6C) and improved hyperglycaemia, glucose intolerance and insulin resistance (Figure 6D‒F). Consistent with the effects of systemic *Ccr1* ablation, adipocyte‐specific *Ccr1* deficiency significantly increased VO_2_ (Figure S10C) and promoted energy expenditure (Figure 6G,H) and adaptive thermogenesis (Figure 6I), whereas VCO_2_ remained similar to *Ccr1^fl/fl^* mice (Figure S10D). Thus, in adipocytes, *Ccr1* deficiency enhances energy expenditure, thereby contributing to the attenuation of obesity and improvement of metabolic parameters.

In line with the increased energy expenditure, adipocyte‐specific *Ccr1* deficiency upregulated UCP1 protein expression in white and brown fat depots (Figures 6J,K and S10E,F). This upregulation coincided with enhanced cAMP‐associated signalling, marked by increased PKA‐C and CREB phosphorylation in both fat types (Figures 6J,K and S10E,F). Consistently, thermogenic markers were also upregulated in eWAT of *Ccr1^AKO^* mice (Figure S10G). Furthermore, adipocyte‐specific *Ccr1* ablation markedly reduced F4/80^+^ macrophage accumulation and the prevalence of CD11c^+^ M1‐like cells, while increasing CD163^+^ M2‐like macrophages (Figure 6L). Thus, adipocyte‐specific *Ccr1* deletion potentiates thermogenic adipose activation via the cAMP/PKA/CREB axis, which in turn counteracts obesity and insulin resistance in the context of HFD feeding.

### 4.7 CCR1 blockade with BX471 prevented obesity development in HFD‐fed mice

**FIGURE 7:**
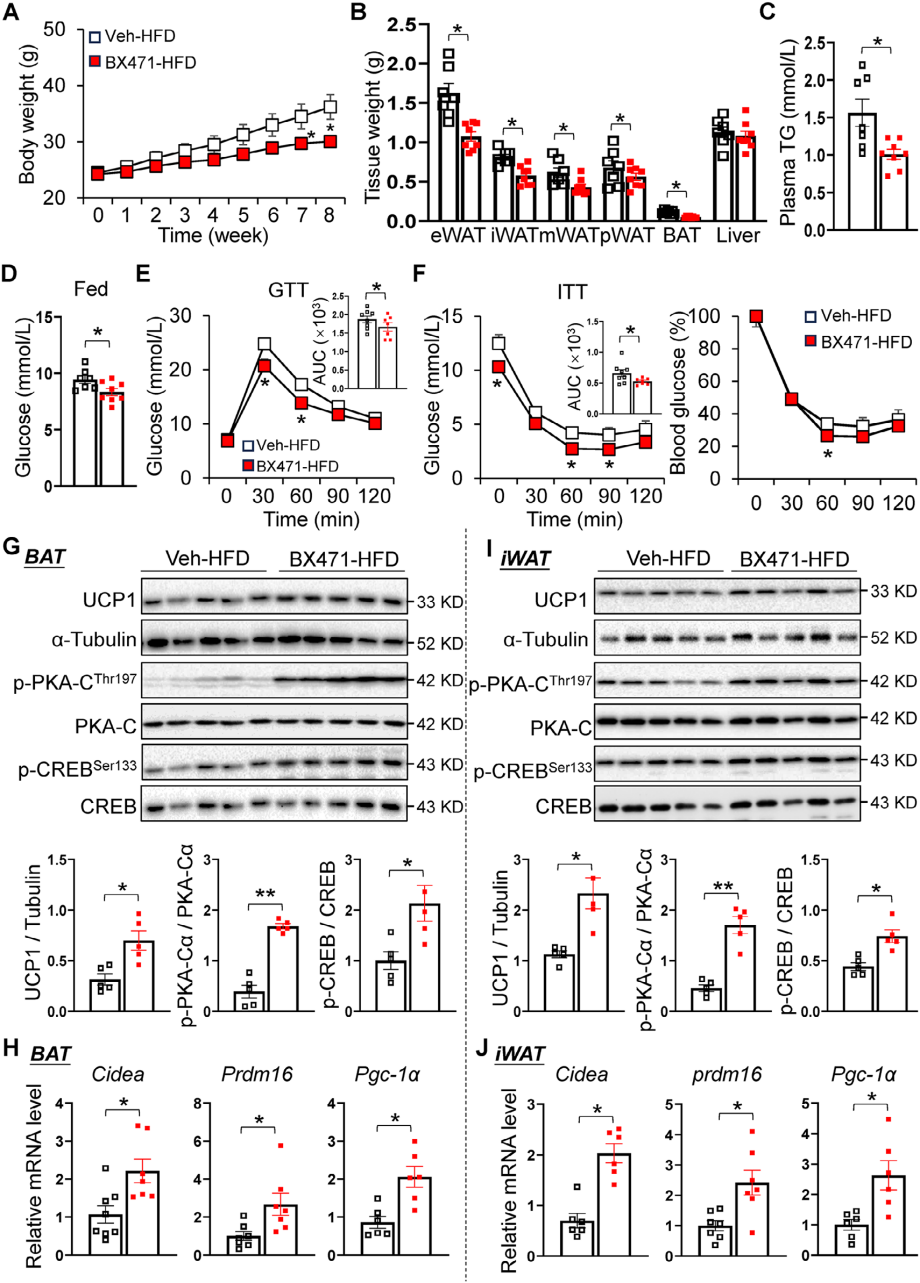
Inhibition of CCR1 with BX471 attenuates obesity development in high‐fat diet (HFD)‐fed mice. (A and B) Body and tissue weights of HFD‐fed mice (*n* = 6‒8). (C) Plasma triglyceride (TG) contents of mice (*n* = 6‒8). (D) Fed blood glucose levels of mice (*n* = 6‒8). (E and F) Glucose and insulin tolerance tests of HFD‐fed mice (*n* = 6‒8). (G) Immunoblots of uncoupling protein 1 (UCP1), p‐PKA‐C and p‐CREB and their total proteins in brown adipose tissue (BAT) of HFD‐fed mice (*n* = 5). (H) Relative mRNA expression of thermogenic genes in the BAT of mice (*n* = 6‒8). (I) Immunoblots of UCP1, p‐PKA‐C and p‐CREB and their total proteins in inguinal white adipose tissue (iWAT) of HFD‐fed mice (*n* = 5). (J) Relative mRNA expression of brown‐related genes in the iWAT of mice (*n* = 6‒8). Data are presented as mean ± SEM. (A‒F, H, J) Two‐tailed Student's *t*‐test. ^*^ *p* < .05, ^**^ *p* < .01.

In a preventive model, 8 weeks of BX471 treatment reduced body weight, fat mass, and plasma TG in HFD‐fed WT mice (Figure 7A‒C), with no detectable effects on *Ccr1* mRNA expression and food consumption (Figure S11A,B). Concomitantly, BX471 reduced blood glucose levels, and ameliorated glucose intolerance and insulin resistance, with lower baseline blood glucose levels observed in BX471‐treated mice compared with vehicle controls (Figure 7D‒F). Moreover, BX471 increased UCP1 protein expression and upregulated brown‐fat‐associated gene expression in both BAT and WAT (Figures 7G‒J and S11C,D), accompanied by enhanced cAMP‐associated signalling activation, as evidenced by increased PKA‐C and CREB phosphorylation in both adipose depots (Figures 7G,I and S11C). Consistently, similar activation of PKA/CREB signalling was observed in 3T3‐L1 adipocytes (Figure S12A).

Furthermore, BX471 administration significantly suppressed the expression of proinflammatory cytokines/chemokines (Figure S13A), reduced infiltration of F4/80^+^ macrophages and induced a change in macrophage polarisation away from the proinflammatory phenotype (Figure S13B,C) in iWAT of HFD‐fed mice. In parallel, inflammation and macrophage infiltration were also attenuated by BX471 in eWAT (Figure S13D,E). Additionally, BX471 alleviated hepatic steatosis and inflammation in HFD‐fed mice (Figure S14A–D). Thus, pharmacological CCR1 blockade coordinately activates fat browning and thermogenesis via cAMP/PKA/CREB signalling, an effect accompanied by alleviation of obesity‐associated inflammation in liver and adipose tissues.

## 5 DISCUSSION

Our findings identify CCR1 as a regulator of insulin resistance, energy homeostasis and inflammation in the context of obesity. *CCR1* expression was augmented in adipose tissue of both obese mice and human individuals; however, the available human datasets lacked male participants, which should be considered when interpreting the translational relevance. Deletion of *Ccr1*, either systemically or specifically in adipocytes, led to a marked reduction in body weight and ectopic lipid content. Moreover, *Ccr1* deficiency promoted a metabolic shift towards increased fat utilisation and enhanced energy expenditure, concomitant with elevated UCP1 expression in BAT as well as WAT, indicative of enhanced thermogenic capacity and adipose browning. Mechanistically, *Ccr1* deficiency potentiated cAMP‐dependent PKA/CREB signalling, providing a molecular basis for CCR1‐mediated regulation of thermogenic programming. Additionally, *Ccr1* knockout mice exhibited improved insulin sensitivity along with diminished adipose tissue inflammation, associated with decreased macrophage infiltration and a shift towards an alternatively activated macrophage phenotype in WAT. Notably, pharmacological inhibition of CCR1 using BX471 largely recapitulated these metabolic phenotypes shown by *Ccr1^‒^* ^/^ *^‒^* mice; thus, CCR1 inhibition may represent a potential target for alleviating obesity‐related metabolic impairment in mice.

Given that UCP1 expression is highly stage‐dependent during DIO, early‐ to mid‐stage HFD feeding may preserve or even enhance BAT thermogenic gene activity as an adaptive response to nutrient excess and sympathetic activation.^26^, ^27^, ^28^ In the present study, the 8‐week HFD model represents an early‐ to mid‐stage obesity state, which may precede the severe mitochondrial dysfunction and thermogenic suppression observed in chronic obesity. Consistent with this concept, we observed elevated UCP1 expression in BAT, together with upregulation of key thermogenic regulators, suggesting that the observed phenotype reflects preserved BAT thermogenic competence rather than pathological suppression.

Cold exposure triggers norepinephrine release from the sympathetic nervous system, which acts primarily on adipocyte β3‐adrenergic receptors to drive BAT activity and WAT browning.^29^ This receptor engagement stimulates adenylyl cyclase, boosting cAMP and subsequently activating PKA. In turn, PKA facilitates lipolysis, generating free fatty acids that fuel thermogenesis and modulate UCP1 activity.^29^ Concurrently, PKA‐dependent phosphorylation of CREB and/or p38 MAPK initiates a transcriptional response featuring UCP1 upregulation.^30^ Thus, these events position the cAMP‒PKA axis as a key driver of reprogramming in adipose tissue.

cAMP is generated when extracellular stimuli, such as neurotransmitters or chemokines, bind to seven‐transmembrane GPCRs coupled to stimulatory G‐protein α‐subunits (Gαs). Upon ligand binding, GDP is exchanged for GTP on Gαs, leading to the dissociation of Gαs from the βγ subunit complex.^31^ The liberated Gαs subunit then activates adenylyl cyclase, which converts ATP into cAMP and pyrophosphate.^32^ In contrast, the Gαi subunit suppresses adenylyl cyclase and thus cAMP production.^33^ Notably, CCR1 signalling couples to Gαi proteins; thus, ligand engagement of CCR1 is expected to attenuate cAMP production and downstream cAMP‒PKA pathway activation.^14^, ^34^ Accordingly, the increased cAMP‐PKA signalling observed in the iWAT of *Ccr1^‒^* ^/^ *^‒^* mice is consistent with the loss of this inhibitory input. RNA‐seq analysis revealed that cAMP‐related signalling was activated in iWAT. Supporting this notion, absence of *Ccr1* increased CREB phosphorylation but decreased p38 MAPK phosphorylation, suggesting that CCR1 regulates adipose thermogenic activity, at least in part, through modulation of cAMP/PKA‐dependent CREB signalling. Of note, the bulk RNA‐seq approach does not resolve cell‑type‑specific contributions; this limitation could be addressed by single‑cell sequencing in future studies.

Additionally, cold‐induced upregulation of *Ccr1* in whole adipose tissues does not necessarily reflect its functional activity within thermogenic adipocytes. CCR1 is expressed not only in adipocytes but also in SVF cells, particularly macrophages, whose composition undergoes dynamic remodelling during cold exposure.^13^, ^21^ Therefore, the increased *Ccr1* mRNA likely reflects immune cell‐associated transcriptional changes rather than enhanced inhibitory signalling in beige adipocytes. In parallel, UCP1 induction represents a cell‐intrinsic thermogenic response in adipocytes driven by sympathetic cAMP signalling and is not directly coupled to bulk tissue *Ccr1* expression.

Proinflammatory signals regulate thermogenesis in brown and beige adipocytes; in obesity, dysregulated signals impair glucose metabolism and disrupt energy homeostasis.^35^ M2 macrophages are known to facilitate the transition of WAT into beige via the production of type 2 cytokines.^36^ M2 macrophage polarisation triggered by chronic cold exposure, along with macrophage‐derived cytokine signalling in both WAT and BAT, is essential for regulating energy expenditure and fatty acid metabolism. Conversely, disruption of M2‐associated cytokine signalling, including the IL‐4/IL‐13 pathway and tyrosine hydroxylase signalling, impairs cold‐induced beige adipogenesis.^37^ CXCL14 produced by BAT recruits M2 macrophages and promotes energy metabolism and browning.^12^ In contrast, CCR5/CCL5 signalling suppresses thermogenic activation and promotes inflammatory remodelling of adipose tissue, thereby acting as a negative regulator of adipose browning.^13^ *Ccl2* deficiency similarly enhances adipose tissue browning by increasing M2 polarisation.^38^ These findings highlight a complex chemokine network that differentially regulates adipose tissue remodelling and thermogenesis.

Extending this chemokine‐centric view, we previously found that *Ccr1* knockout shifts macrophages towards an M2 phenotype by attenuating mTORC1 activation, thereby reducing inflammation and hepatic steatosis.^15^ The present study demonstrates that *Ccr1* deficiency similarly promotes M2 polarisation in adipose tissue, subsequently enhancing adipocyte thermogenic activation. Likewise, our in vitro results showed that IL‐4‐stimulated macrophages lacking *Ccr1* produce paracrine signals that potentiate CREB phosphorylation and UCP1 expression in adipocytes. These findings align with the established role of M2 macrophages in promoting beige adipogenesis through paracrine signalling,^39^, ^40^ together indicating that *Ccr1* deficiency enhances adipocyte browning, at least partially, by promoting M2 macrophage polarisation.

Mechanistically, the M2‐polarising effect of *Ccr1* deficiency may be linked to cAMP signalling, a key intracellular mediator of macrophage reprogramming. Recent findings indicate that elevated cAMP promotes M2‐like macrophage polarisation via a PKA/CREB‐dependent pathway, independent of IL‐4/STAT6 signalling.^41^ This notion is reinforced by observations that cAMP elevation enhances IL‐4‐driven M2 marker expression through a PKA‐C/EBPβ‒CREB pathway. In line with the established role of cAMP in promoting inflammatory resolution,^42^ the cAMP analogue db‐cAMP has been shown to modulate monocyte recruitment along the CCL2/CCR2 axis and to drive M2‐like polarisation, either independently or in combination with interferon‐γ (IFN‐γ)/LPS stimulation, while also amplifying the IL‐4‐induced M2 polarisation in vitro.^43^ By contrast, PKA inhibition impairs db‐cAMP‐induced M2 polarisation and its anti‐inflammatory effects in acute inflammatory models.^43^ Therefore, in addition to inhibiting the mTORC1‐related pathway, *Ccr1* deficiency may promote M2 macrophage polarisation in WAT through increased cAMP‒PKA signalling.

Importantly, pharmacological inhibition of CCR1 with BX471 closely recapitulated the metabolic benefits observed in *Ccr1*‐deficient mice, including reduced adiposity, improved insulin sensitivity and enhanced thermogenic gene expression, and these improvements correlated with cAMP/PKA/CREB pathway activation and reduced adipose inflammation. Unlike CCR2/CCR5‐targeted agents (e.g., cenicriviroc), which have yielded limited clinical efficacy in NASH trials,^44^ blockade of CCR1 may offer a more effective approach for improving metabolic and inflammatory aspects of obesity. Notably, BX471 exhibited no detectable toxicity in lean mice, further supporting its therapeutic potential.^15^

## 6 CONCLUSION

In summary, our findings show that CCR1 adversely regulates energy metabolism and adaptive thermogenesis. Targeting CCR1, either through genetic ablation or pharmacological blockade, elicits a coordinated thermogenic and anti‐inflammatory response, characterised by enhanced WAT browning, BAT thermogenesis, M2‐like macrophage polarisation, and systemic energy expenditure in vivo. This integrated response appears to be mediated in part by cAMP/PKA/CREB signalling, thereby protecting against obesity and insulin resistance in mice. These results position CCR1 as a candidate target for preventing obesity‐associated metabolic abnormalities. Nevertheless, several key issues remain to be addressed in future investigations. The cell‐type‐specific roles of CCR1 in adipocytes versus macrophages or other non‐adipose populations require further dissection using lineage‐specific models, particularly to clarify the in vivo contribution of macrophage‐derived CCR1 signalling to adipose tissue remodelling and thermogenic regulation. In addition, the clinical translational potential of targeting CCR1 warrants thorough validation, including the pharmacokinetic profile, dose‒response relationship, and off‐target effects of BX471. Furthermore, all in vivo experiments were performed in male mice; whether the observed findings extend to females remains to be determined. Addressing these limitations will strengthen the mechanistic rigor and clinical feasibility of CCR1‐targeting strategies against obesity.

## AUTHOR CONTRIBUTIONS

*Data curation, formal analysis, investigation and methodology*: Suili Cai. *Data curation, investigation and methodology*: Mengchen Ma. *Data curation, formal analysis, investigation and writing–original draft*: Yuqin Zhu. *Investigation and software*: Mengru Shi. *Investigation and methodology*: Xiaoyan Dai. *Data curation and formal analysis*: Siyong Zhang. *Data curation, investigation and methodology*: Yanwen Yu. *Formal analysis and software*: Yajiao Wang. *Conceptualisation, investigation, project administration, resources, supervision and writing—review and editing*: Liang Xu.

## CONFLICT OF INTEREST STATEMENT

The authors declare they have no conflicts of interest.

## ETHICS STATEMENT

All animal procedures were performed in accordance with the ethical policies and protocols approved by the Institutional Animal Care and Use Committee (IACUC) of Wenzhou Medical University, China (approval no. wydw2019‐0945).

## Supporting information



Supporting Information

## Data Availability

Sequencing data generated in this study have been deposited in the GEO database under accession number GSE274417. All data supporting the findings of this study are available within the article and its Supporting Information. Additional information or materials related to this study are available from the corresponding author upon reasonable request.
