# Supplementary material for: Chemokine receptor CCR1 restrains adipose tissue remodelling via cAMP/PKA/CREB signalling and adipocyte‒macrophage crosstalk in mice
Source: Clin Transl Med. 2026 Aug 6;16(8):e70762. doi: 10.1002/ctm2.70762 (PMC13444349; doi:10.1002/ctm2.70762)
Supplement: Supplementary file 1 [file CTM2-16-e70762-s001.docx]

**Chemokine receptor CCR1 restrains adipose tissue remodeling via cAMP/PKA/CREB signaling and adipocyte-macrophage crosstalk in mice**

Short title: *Ccr1* deficiency prevents obesity via fat browning

Suili Cai ^1,#^, Mengchen Ma ^1,#^, Yuqin Zhu ^1,#^, Mengru Shi ^1^, Xiaoyan Dai ^1^, Yujie Wang ^1^, Mengru Li ^1^, Yanwen Yu ^1^, Yajiao Wang ^1^, and Liang Xu ^1,^*

^1^ Key Laboratory of Laboratory Medicine, Ministry of Education, Zhejiang Provincial Key Laboratory of Medical Genetics, Cixi Biomedical Research Institute, School of Laboratory Medicine and Life sciences, Wenzhou Medical University, Wenzhou, 325035, China

^#^ These authors contributed equally to this work.

* Corresponding author:

Liang Xu, Ph.D.,

School of Laboratory Medicine and Life Sciences,

Wenzhou Medical University, Wenzhou 325035, Zhejiang, China.

Phone Number: +86-15906495361;

E-mail: [liangxu1023@gmail.com](mailto:liangxu1023@gmail.com)

**Supplementary Information**

**Supplementary Tables**

**Supplementary Table 1–Antibodies used in FACS analysis.**

| Antibody |  |
| --- | --- |
| PerCP-Cyanine5.5-conjugated NK1.1 | eBioscience |
| PerCP-Cyanine5.5-conjugated CD3 | eBioscience |
| PerCP-Cyanine5.5-conjugated CD19 | eBioscience |
| PerCP-Cyanine5.5-conjugated TER-119 | eBioscience |
| allophycocyanin (APC)-eFluor 780-conjugated CD45 | eBioscience |
| phycoerythrin (PE)-conjugated CD11c | eBioscience |
| eFluor 450-conjugated Ly-6G (Gr-1) | eBioscience |
| APC-conjugated CD8 | eBioscience |
| eFluor 450-conjugated CD3 | eBioscience |
| PE-conjugated NK1.1 | eBioscience |
| fluorescein isothiocyanate (FITC)-conjugated CD45 | eBioscience |
| PE/Cy7-conjugated F4/80 | Biolegend |
| Alexa Fluor 647-conjugated CD206 | Biolegend |
| APC-conjugated Ly-6C | Biolegend |
| PE Texas Red-conjugated CD4 | Abcam |
| APC-Cy7-conjugated CD11b | BD Bioscience |
| PE-Texas Red-conjugated CD11b | Invitrogen |

**Supplementary Table 2–Primer sequences.**

| Gene | 5’ Primer | 3’ Primer |
| --- | --- | --- |
| *Acc* | TGGAGAGCCCCACACACA | TGACAGACTGATCGCAGAGAAAG |
| *Arg1* | CTCCAAGCCAAAGTCCTTAGAG | AGGAGCTGTCATTAGGGACATC |
| *Ccl5* | TGCCCTCACCATCATCCTCACT | GGCGGTTCCTTCGAGTGACA |
| *Ccr2* | ATTCTCCACACCCTGTTTCG | GATTCCTGGAAGGTGGTCAA |
| *Ccr1* | CTCATGCAGCATAGGAGGCTT | ACATGGCATCACCAAAAATCCA |
| *Cd11c* | AAAATCTCCAACCCATGCTG | CACCACCAGGGTCTTCAAGT |
| *Cd206* | CAAGGAAGGTTGGCATTTGT | CCTTTCAGTCCTTTGCAAGC |
| *Cidea* | ATCACAACTGGCCTGGTTACG | TACTACCCGGTGTCCATTTCT |
| *Cpt-1α* | AAACCCACCAGGCTACAGTG | TCCTTGTAATGTGCGAGCTG |
| *Dio2* | CAGTGTGGTGCACGTCTCCAATC | TGAACCAAAGTTGACCACCAG |
| *F4/80* | CTTTGGCTATGGGCTTCCAGTC | GCAAGGAGGACAGAGTTTATCGTG |
| *Fas* | AGAGACGTGTCACTCCTGGACTT | GCTGCGGAAACTTCAGAAAAT |
| *Fabp4* | TGAAATCACTCCAGATGACAGG | TCAATATCCCTTGGCTTATGCT |
| *Il-10* | GCTCTTACTGACTGGCATGAG | CGCAGCTCTAGGAGCATGTG |
| *Il-1β* | CTGAACTCAACTGTGAAATGCCA | AAAGGTTTGGAAGCAGCCCT |
| *Ccl2* | AGGTCCCTGTCATGCTTCTGG | CTGCTGCTGGTGATCCTCTTG |
| *Pgc-1α* | ATGTGTCGCCTTCTTGCTCT | ATCTACTGCCTGGGGACCTT |
| *Pparα* | GAGGGTTGAGCTCAGTCA GG | GGTCACCTACGAGTGGCATT |
| *Pparγ* | GTACTGTCGGTTTCAGAAGTGCC | ATCTCCGCCAACAGCTTCTCCT |
| *Prdm16* | GGCGAGGAAGCTAGCCAAA | GGTCTCCTCCTCGGCACTCT |
| *Scd1* | CATCATTCTCATGGTCCTGCT | CCCAGTCGTACACGTCAT TTT |
| *Tnfα* | AAGCCTGTAGCCCACGTCGTA | GGCACCACTAGTTGGTTGTCTTTG |
| *Ucp1* | ACTGCCACACCTCCAGTCATT | CTTTGCCTCACTCAGGATTGG |
| *β-actin* | AGGCCCAGAGCAAGAGAGGTA | GGGGTGTTGAAGGTCTCAAACA |
| *18S* | GGAGAACTCACGGAGGACGAGTA | CCAGTGGTCTTGGTGTGC TGA |

**Supplementary Figures**

**Figure S1. CCR1 is upregulated in adipose tissues of humans and mice with obesity.**

A, B. *CCR1* expression analysis in subcutaneous WAT of obese and lean females using GEO datasets (GSE110729 and GSE25402) from the NCBI database.

C. Relative Ccr1 mRNA levels in adipose tissues of NC- or HFD-fed mice (n = 6-8).

D. Expression profiling of chemokine receptors (*CCR2* and *CCR5*) and CCR1 ligands (*CCL3*, *CCL5*, and *CCL23*) in subcutaneous WAT from the GSE25402 dataset.

E. Relative mRNA expression of *Ccr2*, *Ccr5*, *Ccl3* and *Ccl5* in eWAT of NC- or HFD-fed mice (n = 6-8).

F. Relative Ccr1 mRNA levels in primary adipocytes and SVF from eWAT of NC- or HFD-fed mice (n = 4-6).

G. Immunofluorescence staining for F4/80 (green) and CCR1 (red) in eWAT of HFD-fed mice (n = 3). Scale bars = 25 μm.

Data are presented as mean ± SEM. Statistical analysis: two-tailed student’s *t*-test for A-C, F and G; two-way ANOVA for D. **P* < 0.05, ***P* < 0.01.

**Figure S2. *Ccr1* deficiency slightly attenuates adiposity in NC-fed mice.**

A. Validation of *Ccr1* knockout efficiency by RT-qPCR, showing *Ccr1* mRNA levels in *Ccr1*^-/-^ and WT mice (n = 4).

B. Tissue weights of NC-fed mice (n = 6-8).

C, D. H&E-stained iWAT and BAT sections of NC- or HFD-fed mice (n = 6-8). Scale bars = 100 μm.

E. Glucose tolerance tests in NC-fed mice (n = 6-8).

F. Insulin tolerance tests in NC-fed mice. Blood glucose levels are presented as absolute values and normalized to baseline (n = 6-8).

G, H. Immunoblot analysis of AKT phosphorylation in liver and eWAT under basal conditions (n = 3-4).

Data are presented as mean ± SEM. Statistical analysis: two-tailed student’s *t*-test for A-B and E-H; two-way ANOVA for C-D. ***P* < 0.01, ^##^*P* < 0.01.

**Figure S3. *Ccr1* deficiency increases VO₂ and VCO₂ in NC-fed mice.**

A, B. VO₂ and VCO₂ of NC-fed mice (n = 6).

C. Respiratory exchange ratio (RER) of NC-fed mice (n = 6).

D. Rectal temperature of NC-fed mice (n = 6).

Data are presented as mean ± SEM. Statistical analysis: two-tailed student’s *t*-test for A-D. ^#^*P* < 0.05, ^##^*P* < 0.01.

**Figure S4. *Ccr1* deficiency increases fat browning in HFD-fed mice.**

A. Quantification of immunoblots of UCP1 in BAT (n = 6-8).

B. Relative mRNA levels of thermogenic genes in BAT (n = 6-8).

C. Quantification of immunoblots of UCP1 in iWAT (n = 6-8).

D, E. Relative mRNA levels of brown-related genes in iWAT and eWAT (n = 6-8).

F. Oil Red O staining of adipocytes (n = 4). Scale bars = 200 μm.

G. Relative mRNA levels of thermogenesis- and lipid synthesis-related genes in adipocytes (n = 5-6).

Data are presented as mean ± SEM. Statistical analysis: two-way ANOVA for A-E; two-tailed student’s *t*-test for F-G. **P* < 0.05, ***P* < 0.01; ^#^*P* < 0.05, ^##^*P* < 0.01.

**Figure S5. *Ccr1* deficiency increases activation of the cAMP/PKA/CREB pathway in adipocytes.**

A. Volcano plots of RNA-seq in iWAT of HFD-fed mice (n = 3).

B. GO enrichment analysis of DEGs from RNA-seq data of iWAT in HFD-fed mice (n = 3).

C. Heatmap of cAMP pathway-associated DEGs in iWAT of HFD-fed mice (n = 3).

D, E. Quantification of p-PKA-C and p-CREB in BAT and iWAT of mice (n = 6-8).

F. Relative mRNA levels of thermogenic genes in 3T3-L1 adipocytes (n = 4).

Data are presented as mean ± SEM. Statistical analysis: two-way ANOVA for D-E; one-way ANOVA for F. **P* < 0.05, ***P* < 0.01; ^#^*P* < 0.05, ^##^*P* < 0.01.

**Figure S6. *Ccr1* deficiency enhances thermogenesis in cold-stimulated mice.**

A, B. Quantification of immunoblots of UCP1, p-PKA-C and p-CREB in BAT and iWAT, respectively (n = 4).

C. Relative mRNA expression of thermogenic genes in eWAT of mice (n = 4).

Data are presented as mean ± SEM. Statistical analysis: two-tailed student’s *t*-test for A-C. **P* < 0.05, ***P* < 0.01.

**Figure S7. *Ccr1* deficiency attenuates inflammation in eWAT of HFD-fed mice.**

A. Relative mRNA expression of *Tnfα*, *Ccl2* and *Ccr2* in eWAT of NC- or HFD-fed mice (n = 6-8).

B. Immunoblot analysis of p-P38 MAPK and P38 MAPK in eWAT (n = 6-8).

C. Relative mRNA expression of *F4/80* and *Cd11c* in eWAT (n = 6-8).

D. F4/80-stained eWAT sections (n = 6-8). Scale bars = 100 μm.

E. Relative mRNA expression of *Arg1* and *Cd206* in eWAT (n = 6-8).

Data are presented as mean ± SEM. Statistical analysis: two-way ANOVA for A-D. **P* < 0.05, ***P* < 0.01; ^#^*P* < 0.05, ^##^*P* < 0.01.

**Figure S8. *Ccr1* deficiency promotes M2 macrophage polarization and subsequently enhances adipocyte browning.**

A. TNFα levels in conditioned medium (CM) from LPS-treated peritoneal macrophages isolated from WT and *Ccr1*^-/-^ mice (n = 4).

B. IL-4 levels in CM from IL-4-treated peritoneal macrophages (n = 4).

C. Immunoblot analysis of UCP1 and phosphorylated CREB in primary adipocytes treated with CM derived from IL-4-stimulated peritoneal macrophages (n = 3-4).

Data are presented as mean ± SEM. Statistical analysis: two-way ANOVA for A-C. **P* < 0.05, ***P* < 0.01; ^#^*P* < 0.05, ^##^*P* < 0.01.

**Figure S9. *Ccr1* deficiency attenuates liver steatosis and inflammation in HFD-fed mice.**

A. H&E-stained liver sections of NC- and HFD-fed mice (n = 6-8). Scale bars = 100 μm.

B. Liver TG contents (n = 6-8).

C. Relative mRNA expression of *Fas*, *Scd1*, *Cpt-1α* and *Pparα* in the liver of mice (n = 6-8).

D. F4/80-stained liver sections (n = 6-8). Scale bars = 100 μm.

E. Relative mRNA expression of *F4/80* and *Cd11c* in the liver (n = 6-8).

F. Relative mRNA expression of hepatic *Tnfα* and *Ccl2* (n = 6-8).

G. Immunoblot analysis of p-P38 MAPK, p-ERK and their total proteins in the liver of mice (n = 6-8).

Data are presented as mean ± SEM. Statistical analysis: two-way ANOVA for A-G. **P* < 0.05, ***P* < 0.01; ^#^*P* < 0.05, ^##^*P* < 0.01.

**Figure S10. Adipocyte-specific ablation of *Ccr1* enhances thermogenic activity in HFD-fed mice.**

A. Schematic illustration of adipocyte-specific knockout of *Ccr1* in mice.

B. Validation of tissue specificity showing relative *Ccr1* mRNA levels in peripheral tissues of adipocyte-specific *Ccr1* knockout mice (n = 3).

C, D. VO₂ and VCO₂ of HFD-fed mice with adipocyte-specific *Ccr1* knockout (n = 4).

E, F. Quantification of immunoblots of UCP1, p-PKA-C and p-CREB in BAT and iWAT of HFD-fed mice with adipocyte-specific *Ccr1* knockout (n = 5).

G. Relative mRNA expression of thermogenic genes in eWAT of adipocyte-specific *Ccr1* knockout mice (n = 5).

Data are presented as mean ± SEM. Statistical analysis: two-tailed student’s *t*-test for B and E-G. **P* < 0.05, ***P* < 0.01.

**Figure S11. BX471 increases browning in eWAT of HFD-fed mice.**

A. Relative *Ccr1* mRNA levels in adipose tissues of mice (n = 7-8).

B. Food intake of mice (n = 7-8).

C. Immunoblot analysis of UCP1, p-PKA-C and p-CREB and their total proteins in eWAT of HFD-fed mice (n = 7-8).

D. Relative mRNA expression of brown-related genes in eWAT of mice (n = 7-8).

Data are presented as mean ± SEM. Statistical analysis: two-tailed student’s *t*-test for A-G. *P < 0.05, **P < 0.01.

**Figure S12. BX471 directly increases PKA/CREB signaling activity in 3T3-L1 adipocytes.**

A.	Immunoblot analysis of p-PKA-C, p-CREB and their total proteins in differentiated 3T3-L1 adipocytes treated with BX471 (n = 3).

Data are presented as mean ± SEM. Statistical analysis: two-tailed student’s *t*-test for A. **P* < 0.05.

**Figure S13. BX471 attenuates inflammation of WAT in HFD-fed mice.**

A. Relative mRNA expression of *Tnfα*, *Ccl2*, *Ccr2* and *Ccl5* in iWAT of mice (n = 7-8).

B. F4/80-stained iWAT sections (n = 7-8). Scale bars = 100 μm.

C. Quantification of total macrophages, M1-type, M2-type macrophages, and M1/M2 ratio according to flow cytometry (n = 7-8).

D. Relative mRNA expression of *Tnfα*, *Ccl2*, *Ccl5*, *Ccr2* and *Ccr5* in eWAT of mice (n = 7-8).

E. F4/80-stained eWAT sections (n = 7-8). Scale bars = 100 μm.

Data are presented as mean ± SEM. Statistical analysis: two-tailed student’s *t*-test for A-D. **P* < 0.05, ***P* < 0.01.

**Figure S13. BX471 attenuates hepatic steatosis in HFD-fed mice.**

A. H&E-stained liver sections (n = 6-8). Scale bars = 100 μm.

B. Relative mRNA levels of lipid synthesis-related genes (*Fas*, *Srebp1c*, *Scd1* and *Acc*) and lipidolysis-associated genes (*Cpt-1α* and *Pparα*) in the liver of mice (n = 6-8).

C. F4/80-stained liver sections (n = 6-8). Scale bars = 100 μm.

D. Relative mRNA expression of inflammation-related genes (*Tnfα*, *Ccl2, Ccl5, and Ccr2*) in the liver of mice (n = 6-8).

Data are presented as mean ± SEM. Statistical analysis: two-tailed student’s *t*-test for B and D. **P* < 0.05.
